# Method for and potential value of reflexive stakeholder mapping of a policy evaluation team: the example of the national evaluation of the NHS Pharmacy First scheme

**DOI:** 10.1186/s12961-026-01474-5

**Published:** 2026-03-23

**Authors:** Ayodeji Matuluko, Mirza Lalani, Isaac Yen-Hao Chu, Agata Pacho, Nicholas Mays, Rebecca E Glover

**Affiliations:** Department of Health Services Research and Policy, London School of Hygiene and Tropical Medicine (LSHTM), London, England, UK

**Keywords:** Pharmacy First, Stakeholder analysis, Health policy, Policy evaluation, Reflexivity, Positionality

## Abstract

**Background:**

Pharmacy First (PF) was launched in the National Health Service in England in January 2024, and an evaluation of PF was commissioned by the National Institute for Health and Care Research. The aim of this study was to conduct a reflexive stakeholder analysis of the PF evaluation team and an independent study steering committee (SSC), considering stakeholder positionality and identity.

**Methods:**

All members of the PF evaluation team (including patient and lay members) and the SSC were asked to confirm their affiliations and professional identities potentially relevant to evaluating PF. The data were entered into an online platform for mapping complex systems. Two stakeholder maps were created, showing the connections between both groups and their respective affiliations. Individuals’ positions (i.e. opinions) on PF were also recorded.

**Results:**

All PF evaluation team (*n* = 32) and SSC (*n* = 17) members responded. There were 23 and 33 potentially relevant affiliations reported across the PF evaluation team and SSC, respectively. Across both groups, 25 had more than one affiliation, 24 had a formal health professional identity and 8 held a dual role in public service and academia.

**Conclusions:**

This novel method for reflexive stakeholder analysis of a research team and its SSC has demonstrated that participants span multiple sectors and professional affiliations, but that there was initial potential for imbalance in the SSC. As a result, changes were made, such as the inclusion of two more general practitioners in the SSC, and adapting data collection to include the former. Conducting reflexive stakeholder analysis may be worth consideration by other evaluation teams, in similar contexts, evaluating high profile policy schemes.

**Supplementary Information:**

The online version contains supplementary material available at 10.1186/s12961-026-01474-5.

## Background

Pharmacy First (PF) was launched in England in January 2024 and allows patient referral to community pharmacies for treatment of seven minor illnesses [1]. Following an invitation to tender, the PF evaluation team applied and was successfully commissioned by the National Institute for Health and Care Research (NIHR) to evaluate PF over a 36-month period [2]. PF is a high profile and political priority in England, with significant financial investment into both the policy and the evaluation itself [3]. The PF evaluation team comprises researchers from five UK institutions: London School of Hygiene and Tropical Medicine (LSHTM), University of Nottingham, University of Oxford, UK Health Security Agency (UKHSA) and University of Manchester. The PF evaluation team also includes a Patient and Public Involvement and Engagement (PPIE) group (as lay members). Separate from the PF evaluation team is an independent study steering committee (SSC) mandated by NIHR, comprising academics, general practitioners (GPs), community pharmacy sector representatives, NHS England (NHSE) and UKHSA representatives. The SSC acts in an advisory and oversight role to the PF evaluation team.

In this paper, a novel approach to conducting reflexive stakeholder analysis of the PF evaluation team is presented [2, 4]. For the purposes of this paper, stakeholders are defined as individuals that can exercise influence on the processes of policy implementation and evaluation [5, 6]. Such influences may be attributed to their identities and stances on specific problems, policies and political orientation.

When developing a competitive bid for the PF evaluation, the PF evaluation team ensured a broad composition of the team with diverse expertise across the aforementioned institutions. However, after presenting initial evaluation findings to policymakers, the team was challenged and criticized by one NHSE external stakeholder for being imbalanced. Though policy evaluators expect ad hoc attacks when evaluation findings get sensitive, this was perceived as a strategic risk to future relationships. In response to this external stakeholder’s concern on the composition of the PF evaluation team, a decision was made to analyse the identities and organisational affiliations reflexively to see whether any changes were necessary and feasible.

Stakeholder analysis is usually undertaken to describe the policy positions and resources of the different interest groups involved in a particular policy milieu, but almost always turns a blind eye to the evaluators themselves. In preparation for this internal stakeholder analysis, different stakeholder analysis approaches in literature were assessed, with the discovery that common analyses focused on stakeholder influences and power, as well as stakeholder interest in the issue under study [5, 7–11]. There are gaps in these common approaches, such as the limited consideration of positionality and reflexivity in stakeholder analysis. Positionality pertains to the position or stance a researcher takes in conducting research, through an intentional assessment of their personal characteristics and perspectives in relation to the research question and study population [12]. Reflexivity involves the researcher transparently declaring their assumptions (considering their identity and positionality) and reflecting on how they have conducted research and arrived at conclusions [12]. Evaluator identities and positions can influence every aspect of research, from study design to data collection and data analysis, thus making positionality and reflexivity important [13, 14].

The authors decided to address this gap in the stakeholder analysis literature, and apply concepts from complex systems theory [15, 16]. Complex systems theory was applied to explicate the PF evaluation team members’ roles as evaluators, and further explicate the SSC’s relationships with the PF evaluation team and relevant organizations as well. Since this study drew on stakeholder analysis and complex systems theory, the term “stakeholders” is adopted for this paper to mean the core PF evaluation team (i.e. researchers from LSHTM and the aforementioned collaborating institutions, along with members of the PPIE group) and members of the independent SSC. Analysing both the PF evaluation team and the SSC separately allowed a comparison of the representation across both groups, and afforded the opportunity to highlight the relationships between them [17].

The aim of this study was to conduct a reflexive stakeholder analysis of the PF evaluation team and the SSC. The process of conducting the stakeholder analysis was deemed helpful to the team to understand a priori individual and collective prior assumptions and biases with respect to PF, thereby being able to take this into account when analysing and interpreting the data from the PF evaluation. This paper describes and analyses the identities and the connections of team members with the wider PF policy community of interest. In addition, as an important part of reflexivity in qualitative research, this approach ensures early considerations of positionality [13, 18].

## Methods

The stakeholder analysis was divided into two parts: (1) the PF evaluation team (including the PPIE members), and (2) the SSC. Between October 2024 and April 2025, members of both groups completed tables with their names, confirming their primary and secondary affiliations and professional identities potentially relevant to PF and the evaluation. Primary affiliations included members’ major academic titles or main job titles; secondary affiliations also included academic or job titles that members held in addition to their main role but were either honorary/unpaid or represented the minority of their working time. Members were also asked to list their professional identities, and while it was expected that those with clinical affiliations or backgrounds would declare these, this was left open to interpretation and members were free to provide non-clinical identities such as health services researcher, for example. The PF evaluation team table did not gather data on conflicts of interest (COIs), while the SSC table did, because COIs in the study team were considered by NIHR as a part of awarding the bid and no precluding COIs were declared. A separate project manager directly liaised with SSC members to fill in their information in the table, and cross-checked information reported by SSC members. All affiliations reported by members of both the PF evaluation team and the SSC were further double-checked by A.M. (first author and a researcher at LSHTM) from publicly available information contained in websites (institutional). The data gathered from the PF evaluation team and the SSC were organized by A.M. in Microsoft Excel^®^ and entered into Kumu^®^ [19], an online platform for mapping systems and networks which has been used by different researchers to document and visualize stakeholder mapping [16, 20–23]. Two separate stakeholder maps were created, one of the PF evaluation team and one of the SSC.

For each potentially relevant organisation with which the PF evaluation team and SSC were affiliated, the organizations’ formal, publicly available (until September 2025 – via websites, news articles or press releases) positions on the PF policy, if any, were analysed.

## Results

All 32 (100%) evaluation team and all 17 (100%) SSC members responded.

### PF evaluation team characteristics

**Fig. 1:**
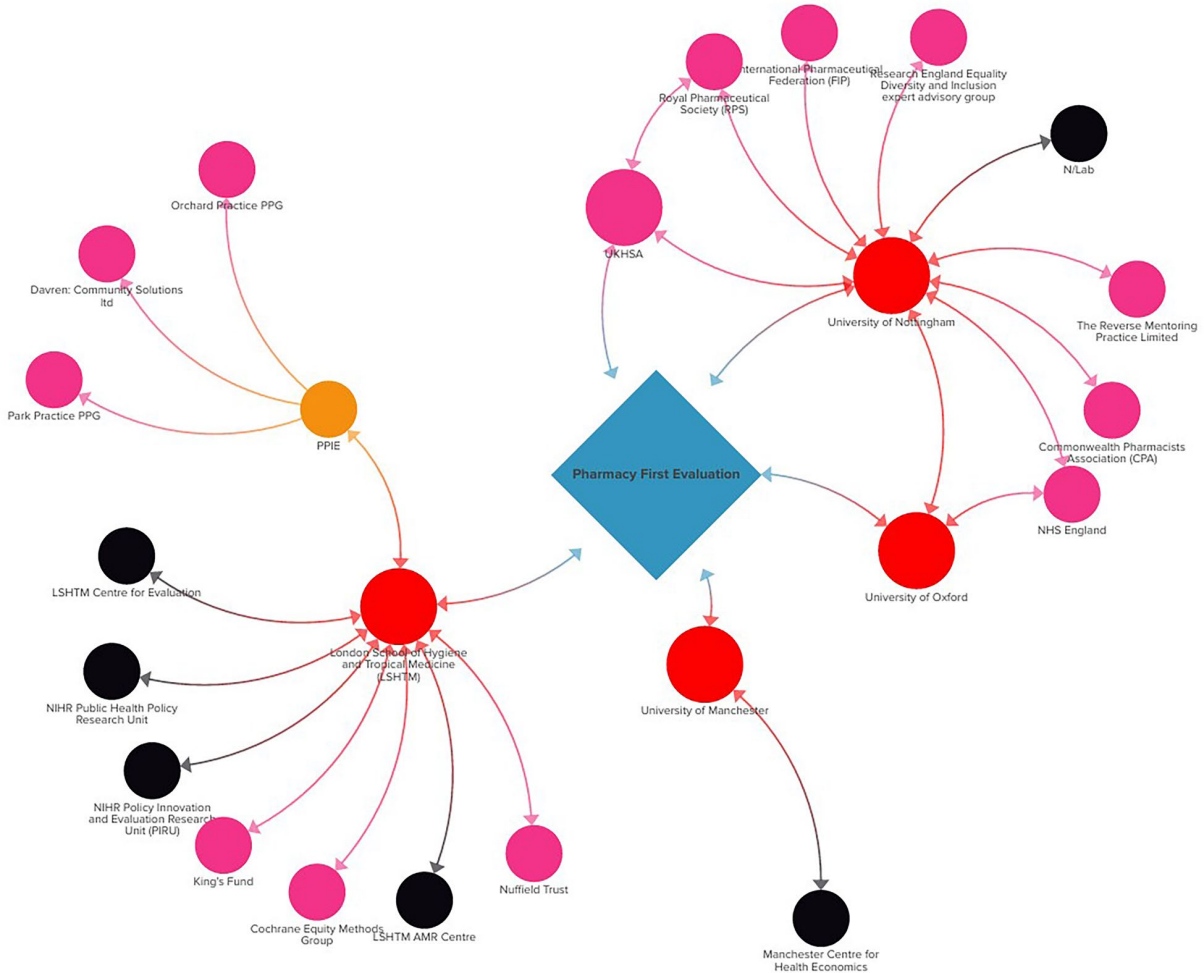
Stakeholder Map of the Pharmacy First (PF) evaluation team’s affiliations relevant to PF

The PF evaluation team reported 23 affiliations, across government agencies (e.g. NHSE, UKHSA), professional bodies (e.g. the Royal Pharmaceutical Society), academic institutions (LSHTM, etc.) and PPIE organisations. Figure 1 is the visualisation of the the PF evaluation team members’ reported institutional affiliations/relationships potentially relevant to the evaluation.

In total, 13 (41%) of the PF evaluation team members reported more than one affiliation. Overall, 16 different professional identities were reported, with 11 (34%) reporting a formal health professional identity (i.e. doctor, nurse, pharmacist). Furthermore, nine (28%) members listed more than one identity. Four (13%) reported holding a dual role in a government agency and academia. These results are outlined in Table 1 and Fig. 1.

**Table 1:** Professional identities of members of the PF evaluation team

Professional role	Number of members on the PF evaluation team
Pharmacist	8*
Health services researcher	5^†^
Health economist	5
Epidemiologist	3
Medical doctor	2^*‡*^
Public health researcher	2^*§*^
Implementation science researcher	2
Medical statistician	1
Statistician	1
Nurse	1
Sociologist	1
Health researcher	1
AI/data scientist	1
Qualitative researcher	1
Health policy analyst	1
Clinical research professional	1

*Includes one non-UK registered pharmacist and one formerly practising UK pharmacist

^†^One qualitative health services researcher

^*‡*^One UK General Practitioner

^*§*^One pharmaceutical public health researcher

Overall, members of the PF evaluation team come from diverse professional backgrounds, with more pharmacists (*n* = 8) being represented on the team than any other professional role, although one of the eight pharmacists, who previously worked as a UK pharmacist, declared that they were no longer practising and not currently on the General Pharmaceutical Council register. In addition, a health policy analyst (*n* = 1) reported being involved in pharmacy practice research in the 1990s. Finally, a medical doctor with an NHSE role as National Clinical Director (NCD) for prescribing reported having a policy interest in the PF scheme, although not directly working on it in their NHSE role. The list of affiliations was not meant to be exhaustive; the team have other affiliations which were not judged to be directly pertinent to the Pharmacy First evaluation.

The subject matter of the evaluation necessitates experts in pharmacy and pharmacy practice research, and the PF evaluation team unsurprisingly has a high proportion of pharmacists and PF-related policy roles. The range of expertise shown in Table 1 suggests a team with the skills and experience to undertake a rigorous evaluation of PF, and includes other clinical professionals apart from pharmacy.

### Study steering committee characteristics

The SSC reported 33 affiliations, with affiliations (primary and secondary) spread across institutions within academia (e.g. University College London, University of Bristol, etc.), professional bodies (e.g. Community Pharmacy England, Community Pharmacy Scotland), Government agency (e.g. NHSE), the private pharmacy sector (e.g. service providers such as Boots^®^) and the charity sector (e.g. Sutton Older Peoples Welfare).

**Fig. 2:**
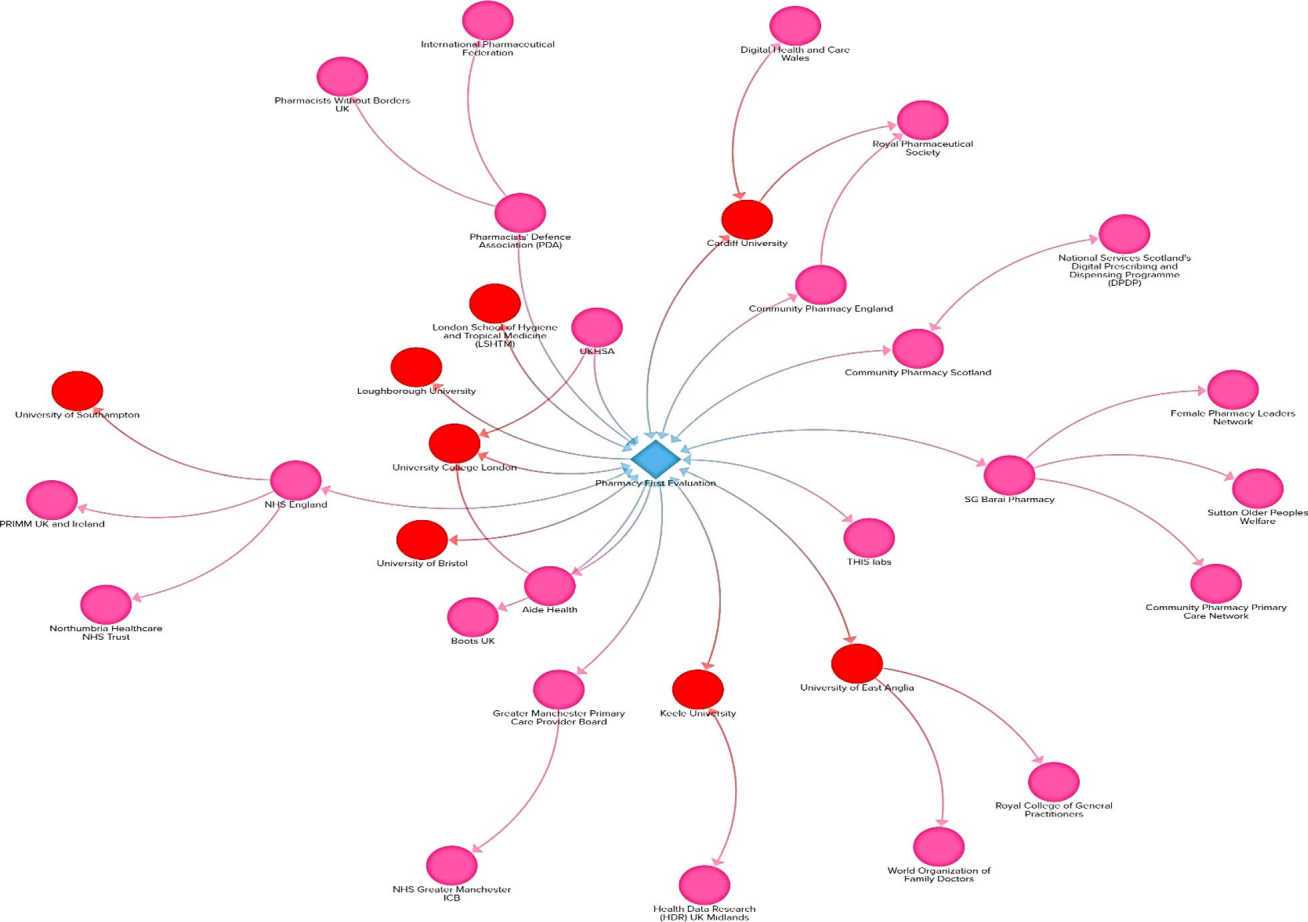
Stakeholder map of the PF study steering committee

In total, 12 of the 17 (71%) SSC members reported more than one affiliation. Thirteen (77%) had a formal health professional identity (doctor or pharmacist) and four (24%) reported holding a dual role in a government agency and academia (with one mentioning their involvement in the co-design of the PF clinical pathways for seven common conditions and antimicrobial resistance [AMR] risk mitigation strategy). Other reported professional identities or fields include Chief Medical Advisor/Public Health/Consultant in Infectious Diseases and Microbiology, Professor of Biostatistics, Professor of Sociology and Professor of Health Economics and Policy. Figure 2 shows the visualization of the stakeholders in the SSC and their reported institutional affiliations/relationships.

### Positions of stakeholders’ affiliated organisations on Pharmacy First

As shown in Table 2, out of 46 organisations listed by PF evaluation team and SSC members, eight (17%)—with seven being pharmacy related organisations—had a positive position on PF (i.e. positive views about the PF). Thirty-seven (80%) had no official position on PF and one (2%) organisation had mixed views on PF. In total, 13 out of 49 (27%) members across the PF evaluation team and SSC were affiliated to seven pharmacy related organizations with positive positions. One (2%) member reported affiliation to an organization (Royal College of General Practitioners, RCGP) with a mixed view of PF. A total of 45 out of 49 (92%) members on the PF evaluation and SSC teams were additionally affiliated to organizations with no official positions on PF.

**Table 2:** Stakeholder organizations’ positions on Pharmacy First in England (affiliations reported by the PF evaluation team and SSC)

Organization category	Organization name	Positive position	Mixed position	No official position
Public service bodies	NHS England	✓		
	Greater Manchester Primary Care Provider Board			✓
	UK Health Security Agency			✓
	Digital Health and Care Wales			✓
	National Services Scotland’s Digital Prescribing and Dispensing Programme (DPDP)			✓
Professional bodies	Royal Pharmaceutical Society	✓		
	Community Pharmacy England	✓		
	Community Pharmacy Scotland	✓		
	International Pharmaceutical Federation (FIP)	✓		
	Royal College of General Practitioners (RCGP)		✓^¥^	
	General Pharmaceutical Council (GPhC)			✓
Charities and service user groups	Commonwealth Pharmacists Association (CPA)			✓
	Davren: Community Solutions Ltd			✓
	Park Practice PPG			✓
	Orchard Practice PPG			✓
	Sutton Older Peoples Welfare			✓
Special interest groups	Female Pharmacy Leaders Network			✓
	Cheam and Sutton Community Pharmacy Primary Care Network			✓
	Prescribing and Research in Medicines Management (PRIMM) (United Kingdom and Ireland)			✓
Trade unions	Pharmacists’ Defence Association (PDA)	✓		
Service providers	Boots UK	✓		
	SG Barai Pharmacy	✓		
Academic institutions	London School of Hygiene and Tropical Medicine (LSHTM)			✓
	University of Nottingham			✓
	University of Oxford			✓
	University of Manchester			✓
	Loughborough University			✓
	Cardiff University			✓
	Keele University			✓
	University of East Anglia			✓
	University of Bristol			✓
	University College London			✓
	University of Southampton			✓
Other – research centres and think tanks	Nuffield Trust			✓*
	King’s Fund			✓*
	NIHR Policy Innovation and Evaluation Research Unit (PIRU)			✓
	NIHR Public Health Policy Research Unit			✓
	LSHTM AMR Centre			✓
	Cochrane Equity Methods Group			✓
	LSHTM Centre for Evaluation			✓
	Health Data Research UK			✓
	THIS labs			✓
Other	The Reverse Mentoring Practice Limited			✓
	Research England Equality Diversity and Inclusion expert advisory group			✓
	Aide Health			✓
	Pharmacists without Borders (Operational) Limited			✓

^*^Although these organizations do not have a formal position on Pharmacy First (PF), they have received funding from Community Pharmacy England (CPE) for a commissioned review on the future of community pharmacy. CPE itself has a positive view of PF

^¥^Historical views on community pharmacy practice, not specific to PF

While affiliated organisations may not have any influence on individual views, out of an abundance of caution we have reported these.

### Presentation of findings to stakeholders

The findings of this stakeholder mapping process have been presented to a policy advisory group – separate from the NIHR-appointed SSC – which the LSHTM team took the initiative to set up within the first year of the evaluation (in response to the challenge from NHSE about the PF evaluation team and the SSC being insufficiently balanced). The policy advisory group includes policymakers in NHSE and the Department of Health and Social Care (DHSC). These findings have also been included in other external presentations to improve transparency and build trust with wider interest groups.

## Discussion

To the authors’ knowledge, this is the first policy evaluation to set out an evaluation team’s positionality using these methods. This reflexive stakeholder analysis of the PF evaluation team and the SSC has led to a highly transparent account of members’ affiliations and the positions on PF of these organizations. Two stakeholder maps were developed showing the connections between the evaluation team and SSC members, and their respective affiliations, building on stakeholder analysis approaches in literature [5, 7–11]. Despite the core PF evaluation team being formally composed of five institutions, the reflexive stakeholder analysis has revealed that the PF evaluation team and SSC actually span multiple organizations (*n* = 45).

Evaluations, seldom report how professional identities and perspectives may shape the progress and outcomes of the research, with evaluation teams having to walk a tight-rope between accusations of “bias” if too close to the topic, or “inexpert”, if too far removed [11, 24]. In the United Kingdom, independent research is frequently commissioned to evaluate health and care system policies that can be, at times, contentious and/or often vigorously defended by their proponents [25, 26]. Similarly, research studies funded by industry often raise concerns about maintaining independence when the funder is also (indirectly) responsible for policy conception and development, and, in many ways, personally invested to demonstrate adequate return on investment; such motivations can lead to tensions with researchers/evaluators [27]. As such, external evaluations risk poor reception by key stakeholders if findings do not confirm the utility of a programme; sometimes defensive policymakers (owing to possibly being under pressure from ministers) may “shoot the messenger” [24, 27]. Proponents of particular policies are often highly invested in the success of these policies so can find it difficult to accept research and evaluation findings that show the limitations or unintended consequences of policies and can criticize the researchers for lack of understanding of the issues at stake or a biased interpretation of their data [24].

During the course of the evaluation, teams sometimes need to justify their independence and/or integrity rather than spending that time discussing and disseminating findings [28]. Policy evaluators will be very familiar with these less positive responses to evaluation, though this is rarely captured in the formal evaluation literature [24]. It is therefore important for evaluation teams to consider how best to protect their reputations, recognizing that policymakers are often understandably invested in the successes of policies they have helped to develop, and may struggle to come to terms with findings and interpretations that challenge their assumptions and perceptions [24, 28]. The more researchers are aware of their own positionality and the influence of their prior experience on their approach to the research and their interpretation of their findings, the stronger their research is likely to be [24, 29].

In this paper, the organizations have either positive positions, no official positions or mixed positions on the PF service. Positive positions on PF were recorded primarily from pharmacy affiliated organizations and NHSE (Table 2). As advocates and representatives of the pharmacy profession, it is expected that the pharmacy affiliated organizations would have positive positions on PF. With 13 out of 49 (27%) members across the PF evaluation team and SSC being affiliated to the seven pharmacy related organizations (with positive positions), the authors recognize how their inclusion in the team may influence their approach to the study and any concerns they may have about the potential impact of the findings, in terms of their potential inherent biases. However, as shown in Figs. 1 and 2, 45 out of 49 (92%) members on the team are additionally affiliated to organizations with no official positions on PF, or mixed positions. One SSC member reported an affiliation to RCGP, which has historically published positively and negatively on community pharmacy practice, with a published piece welcoming the inclusion of community pharmacists in primary care networks, but also emphasizing the need for limitations of scope within community pharmacy [30].

While not directly represented within the PF evaluation team or the SSC, some other organizations have mixed (including very negative) opinions on PF, with a notable example being the British Medical Association (BMA; a professional body that represents medical doctors in the UK), which, while not being overtly in opposition to the implementation of PF, has raised varying concerns about the impact of PF on GP workflows [31–33]. These concerns include teething issues with digital systems that GP practices use, which pharmacies have access to, to make notes on patient diagnoses, observations and medications. GP representatives have raised concerns about the additional workload created by this, owing to GPs needing to follow-up and ensure continuity of patient care as a result of clinical decisions made by pharmacies via PF [31, 32]. In addition, BMA members and office holders have complained about the level of funding allocated to pharmacies for PF, versus the settlement for general practice in the 2024–2025 funding round, with some individual GPs publicly critical of insufficient funding being allocated to the GP sector [34]. As there are four UK registered GPs on the PF evaluation team (*n* = 1) and SSC (*n* = 3), respectively, and one internationally-accredited medical doctor on the PF team, it is possible that their identity and relationships with the wider GP/medical sector could potentially impact their views of the evaluation (and of the PF service) over time.

Though the PF evaluation team and SSC have been reflecting on their own positions and affiliations, there is also a recognition of the positions in the wider academic field that may also influence PF evaluation team/SSC members and evaluation study participants’ (i.e. community pharmacists, GPs and service users) views, and the decision of policymakers. For example, prominent academic researchers in AMR sent a letter to the Daily Telegraph urging that antibiotics should not be prescribed in PF without a prior diagnostic test [35]. These letters, along with other methods of shifting and influencing public debate, mean that this stakeholder analysis is, in essence, a point-prevalence survey. That is, the affiliations (and the views of those organizations) are only presented at one point in time, since stakeholder organizations’ positions are dynamic and liable to change during the life cycle of PF and its evaluation. Nevertheless, it is considered important to capture these views and work towards iteratively analysing any changes to these views as they evolve during the PF scheme implementation, as they help to understand how the policy landscape has changed over time.

Finally, the external context of the policymakers in NHSE could have an influence on the drive for successful implementation of PF. What with the abolishing of NHSE, announced in March 2025, the policymakers in NHSE would feel increasing pressure to deliver “successful” policies (such as PF) owing to a reduction in staffing numbers and staff having to potentially reapply for their own jobs in the merger with DHSC [36–38].

### Strengths and limitations

There are a number of strengths in this work. The stakeholder analysis approach is transparent. All those directly involved in the PF evaluation were included and required to put forward their potentially relevant affiliations, while also being transparent about their professional identities. Furthermore, the representation of patients and the public through the inclusion of the evaluation’s PPIE group was ensured. During the process of putting together the SSC, it became apparent that there was a case for increasing the involvement of GPs, so two academic GPs were proposed to join the SSC.

Nevertheless, a number of limitations exist in this work. There were slightly unclear boundaries about which affiliations to gather and include in the analysis; i.e. (1) the funding stakeholders receive(d), and (2) the reporting of honorary and historical affiliations versus reporting of primary, current affiliations. PF evaluation team members and the SSC were not asked for their personal views on PF, as that was not the goal of this analysis. It is unclear to what extent the bodies which may issue formal views on health policy influence the views of their membership, or if they simply reflect/mirror the views of their constituents. Also, delineating formal versus informal views proved challenging; it was considered that just because a press release or something with a negative view could not be found, did not mean they did not exist.

In addition, the analysis reported in this study focused on professional identities and affiliations, since it was undertaken in direct response to stakeholder challenge about the professional composition of the PF evaluation team. Thus, the authors did not collect data on members’ gender and race. It is considered best practice under the Caldicott principles (a legal responsibility owing to GDPR regulation), and best practice according to the data minimization theory, to only collect the data required for an intended analysis [39].

Furthermore, while data has been gathered from all members of the PF evaluation team (including the PPIE members) and the SSC, it is recognized that information gathered might be imperfect with information gathering relying on individual reporting and publicly available information. However, this stakeholder analysis approach still goes further than the widely accepted approach of taking a reflexive approach to the analysis and interpretation of data, especially qualitative findings.

### Implications for the evaluation of Pharmacy First

Carrying out this stakeholder analysis revealed that the PF evaluation team and SSC span multiple sectors and professional affiliations, and thus there is an increased need to be conscious of how team members’ identities or expertise may influence evaluation findings. This reflexive stakeholder analysis is considered a useful process in aiding transparency in cases where the PF evaluation team may be accused of bias when more findings develop as the evaluation progresses. The process of carrying out the stakeholder analysis has also allowed the core PF evaluation team to make changes since the evaluation began. For example, following the observation that the team was composed of various pharmacists from different sectors, additional academic GPs were included in the SSC and qualitative data collection was tailored to include interviews with GPs. Thus, it is believed that NHSE’s concerns about evaluation team identities, and the composition of the SSC seem to have abated (at least for the moment). Furthermore, there is a plan to use this stakeholder analysis reflexively throughout the life cycle of the PF evaluation project; some examples of these include presentation of evaluation findings at conferences, and aligned publication strategies across the PF evaluation team (cognisant that different members of the team interpret data in different ways). Thus, this stakeholder analysis is useful to aid continuous sense making across the team and cross-referencing the differences in findings.

## Conclusions

Conducting reflexive stakeholder analysis may be worth consideration by other evaluation teams in similar contexts, evaluating high-profile policy initiatives. The authors note that no positionality or transparency exercises happen within policy teams and so this could be one avenue for improving transparency in the future. Reflexive, internal team analysis could be embedded into the process of stakeholder analysis undertaken when evaluating a new policy or intervention, whether in health services research or any applied evaluation. Furthermore, reflexive stakeholder analysis could be employed by governance committees in academic institutions and even within health systems projects.

This would enable greater consideration of positionality tensions, enable stakeholders to establish balanced, reflexive views on the impact of policy initiatives and better manage policymaker expectations. It may, ultimately, serve to increase trust in public institutions.

## Supplementary Information

Supplementary Material 1.

## Data Availability

All data generated or analysed during this study are included in this published article [and its supplementary information file].

## Abbreviations

AMR: Antimicrobial resistance
BMA: British Medical Association
COIs: Conflicts of interest
CPA: Commonwealth Pharmacists Association
DHSC: Department of Health and Social Care
DPDP: Digital Prescribing and Dispensing Programme
FIP: International Pharmaceutical Federation
GPs: General practitioners
LSHTM: London School of Hygiene and Tropical Medicine
NCD: National Clinical Director
NHS: National Health Service
NIHR: National Institute for Health and Care Research
PDA: Pharmacists’ Defence Association
PF: Pharmacy First
PIRU: Policy Innovation and Evaluation Research Unit
PPG: Patient participation group
PPIE: Patient and Public Involvement and Engagement
PRIMM: Prescribing and Research in Medicines Management
RCGP: Royal College of General Practitioners
RPS: Royal Pharmaceutical Society
SSC: Study steering committee
UK: United Kingdom
UKHSA: UK Health Security Agency
