# Supplementary material for: Method for and potential value of reflexive stakeholder mapping of a policy evaluation team: the example of the national evaluation of the NHS Pharmacy First scheme
Source: Health Res Policy Syst. 2026 Mar 23;24:39. doi: 10.1186/s12961-026-01474-5 (PMC13126774; doi:10.1186/s12961-026-01474-5)
Supplement: Supplementary file 1 — Supplementary Material 1. [file 12961_2026_1474_MOESM1_ESM.docx]

**Supplementary file**

**List of other affiliations of SSC members:**

1. Member on various national boards in Wales impacting on policy decisions
2. NIHR
3. MRC
4. UKRI
5. NICE advisory group – common infection guidelines 2017-2022
6. WHO Advisor for Antimicrobial Resistance and Healthcare associated infections
7. Chair of English Surveillance Programme for Antimicrobial Use and Resistance 2013-2021
8. UK AMR NAP Board
9. NHSE AMR delivery board
10. Newcastle University
11. Leeds University
12. Chair of NIHR Steering Group - NIHR Inequalities in Overprescribing Project Steering Committee
13. Steering Group Member – MULTIPLY AI Polypharmacy Study (Newcastle University)
14. Advisory Group Member – OSCAR study (Oxford University)
15. Chair, NIHR Global Health Research Development Award Funding Committee
16. Advisory Committee, NIHR programme grant ‘C-it, Du-it’, 2022-present
17. Member of NHS England AMR Programme Board and UK AMR Delivery Board.
18. Member of Royal Pharmaceutical Society AMR Expert Advisory Group.
19. Co-investigator on MicroGuide2 study, sponsored by MSD.
20. Regularly asked to submit to parliamentary select committees
21. WHA annual assembly speaker.

**List of funding received by SSC members**

1. Receives part funding from NIHR Applied Research Collaboration West Midlands
2. Grant funding over past 10 years from: UKRI, WHO, Global Fund, FCDO, Wellcome Trust, The Trinity Challenge, MSD for Mothers.
3. THIS Labs is supported by the Health Foundation
4. I have accepted grant funding from MRC, the Health Foundation, NIHR, Australian Heart and Lung Institute, SPCR, Stroke Association.
5. Research funding on infectious diseases, antibiotic use and AMR from NIHR, MRC, UKRI
6. Ad hoc – honoraria for work with drug companies
7. Ad hoc – honoraria for work with drug companies (less than once per year).
